# Conditions Under Which Predispensing of Antiviral Drugs to Individuals at High Risk of Death from Pandemic Influenza Is Expected to Save Lives

**DOI:** 10.1371/currents.RRN1007

**Published:** 2009-08-21

**Authors:** Edward Goldstein, Joel C. Miller, Justin O'Hagan, Marc Lipsitch

**Affiliations:** ^*^Harvard School of Public Health and ^‡^Dept of Epidemiology and Center for Communicable Disease Dynamics, Harvard School of Public Health

## Abstract

In this Knol we consider the net benefits of predispensing antivirals to high-risk individuals during an influenza pandemic, where the measure of the benefit is the number of lives saved by antivirals in the whole population. Predispensing has two potential benefits: first, individuals to whom antivirals have been predispensed may be able to initiate treatment earlier than if they had to wait to obtain and fill a prescription, reducing their risk of progression to severe disease. Second, if demand exceeds supply, predispensing to high-risk individuals increases the chance that the course will be used by a high-risk, rather than a low-risk individual. The disadvantage of predispensing is that a course may be sequestered by a person who does not need it (because s/he does not become infected) or wastes it (using it for something other than influenza). We derive mathematical conditions under which the advantages outweigh the disadvantages and suggest that for individuals at considerably higher than average risk of death from pandemic influenza (for example, greater than approximately 5x the population average risk), predispensing of antivirals up to perhaps 20% of the total antiviral stockpile is very likely to result in a net savings of life. Data to parameterize this model are limited, but we believe such a policy would be robust to a reasonable range of uncertainty, and we note that decisions about predispensing will need to be made before definitive data are available. More precise estimates of the relative risk of death for groups beyond pregnant women are urgently needed for decision making about prioritization of antivirals, vaccines and other countermeasures.

 A key objective for the response to the autumn wave of pandemic influenza A/H1N1 (H1N1pdm) in the Northern Hemisphere is to reduce severe morbidity and mortality that would result from an unmitigated pandemic.  Broadly, such responses may be divided into two groups: (a) efforts to reduce population-wide transmission of the virus and thereby protect individuals from becoming infected, either permanently or for a period of time until vaccines become widely available , and (b) efforts to protect individuals at particularly high risk of complications from becoming infected or, if infected, from developing severe disease.  Here we focus our consideration on the United States, though most of what is said applies more widely. 

Options for the first group of interventions for H1N1pdm are limited in the short term.  Vaccine availability in the US will likely start in September, but it will likely be late 2009 or early 2010 before a substantial fraction of the population can be immunized, most likely by 2 doses of vaccine 3 weeks apart.  In two of the three 20^th^ century pandemics, the peak of the fall wave was in September or October in most places [1], and a repeat of that scenario would mean that vaccines were not available early enough, in large enough numbers, to substantially affect epidemic spread.   Social distancing measures have been to a large extent ruled out in the United States, as exemplified by guidance from the US CDC to try to keep schools open even in the face of ongoing transmission [2].  Antiviral stockpiles in the US are generally judged too small to be used for substantial reductions in transmission, so are mainly being used for treatment and for prophylaxis of health care workers. 

With small amounts of vaccine and antivirals available, it is well understood that they are best used to reduce severe morbidity and mortality in groups at high risk, rather than to try to slow transmission [3] [4].  Pregnant women are at approximately 4-fold higher risk of hospitalization from H1N1pdm than the general population, and they are overrepresented among fatal cases [5].  Other high risk groups have also been identified by health authorities, though the extent of their relative risks has not been published.  Predispensing antiviral drugs to individuals in such groups – so that these medications are available to high-risk persons at the time they first become symptomatic with H1N1pdm infection, without the need to seek a prescription or fill it in a time of possible scarcity – may be a valuable strategy to reduce their risk of severe disease or mortality.  Predispensing here is defined as a policy (most likely implemented by an individual physician) of prescribing and urging a patient to fill the prescription for a course of neuraminidase inhibitor in advance of any known infection with H1N1pdm.  The patient would be instructed to begin self-treatment (possibly following communication with the physician) at early signs of possible H1N1pdm infection.  

Here we define conditions under which predispensing a defined quantity of antivirals, one course each to a defined subset of the population at high risk of death from H1N1pdm infection, would provide a net benefit in terms of reducing total mortality compared to a policy of not predispensing the courses, and leaving them in state and national stockpiles for use by prescription, either by mild outpatient cases or by severe, hospitalized patients.

**Benefits and harms of predispensing**

We assume here that we seek to optimize the number of lives saved (i.e. minimize the number of lives lost) due to H1N1pdm.  For clarity we speak throughout this paper of “lives saved”; however, with suitable modifications to the definitions of all terms, we could equally attempt to prevent hospitalizations or intensive care admissions.   There are at least two possible benefits, and two harms, to predispensing.  The first benefit of predispensing is that a patient possessing a predispensed course of antivirals will likely begin therapy earlier in the course of H1N1pdm infection than one who has not and must therefore acquire a prescription (possibly including a visit to a physician) and fill it, thereby most likely delaying treatment.  This benefit accrues whether or not there is a shortage of antivirals, since it simply reflects the time required to acquire and fill the prescription.  A second possible benefit occurs if the demand for antivirals exceeds supply, so that not all patients who attempt to acquire antivirals can do so.  In this situation, predispensing a course assures that it is in the hands of someone who, if untreated, would be likely to develop severe disease, rather than (potentially) going to someone who will not likely develop severe disease, and whose benefit from taking the antiviral would therefore be less.  

The first harm of predispensing also occurs only if total demand for antivirals exceeds supply, namely, a predispensed course is unavailable to anyone who may need it other than the person who has received it.  If demand exceeds supply, this means one more person, possibly someone who would benefit greatly from having the antiviral, who is unable to obtain it.  An additional harm of predispensing is the possibility that a predispensed course will be used inappropriately (say, to treat non-influenza infection).  This harm is not considered separately in our analysis but is modeled as a reduced benefit of predispensing, as we describe below.

To assess the net benefit or harm from predispensing, we define some notation, summarized in Table 1.  We divide the total population into two groups: a *high-risk group* for which we are considering predispensing, and *the general population*, which is comprised of all individuals not in the high-risk group.  These constitute respectively a proportion *q*  (high risk) and 1-*q*  (general population) of the total population.  Within the general population, risks may vary, so some individuals may be at higher risk than others.   All quantities in our notation are defined as proportion of the total population, hence lie between 0 and 1.  Let *T* be the total supply of antivirals (as a proportion of the population); thus, if enough antivirals are available for treatment of 20% of the population, then *T*=0.2 .  Let *D* be the demand for antivirals in the general population, that is the number of individuals not at high risk who would receive antiviral treatment in the general population if supply were not limited; in practice, some of this demand may be unmet as the supply is constrained.  Let \begin{equation*}p_d\end{equation*} be the probability of dying from the infection over the whole course of the epidemic in the whole population (if no antivirals are used); the death probability in a high risk group is \begin{equation*}Rp_d\end{equation*} .  Note that these death probabilities are not conditional on infection (i.e., are not case-fatality proportions) but are unconditional, reflecting the risk of infection times the risk of dying from infection.  Here the number \begin{equation*}R\end{equation*} is the relative risk of dying in a high risk group compared to the general population. \begin{equation*}R\end{equation*} can be estimated from the existing epidemic data while \begin{equation*}p_d\end{equation*} may be hard to estimate a priori; as we shall see, \begin{equation*}p_d\end{equation*} is factored out of our equations and need not be known.   Let \begin{equation*}p_S\end{equation*} be the probability that a course of antivirals obtained from a stockpile during the epidemic would save a life of a person who would die otherwise.  We assume that this probability is the same between high-risk individuals and members of the general population.  This key assumption may be incorrect, and is discussed later.  Let \begin{equation*}Bp_s\end{equation*} be the probability that a predispensed course of antivirals would save a life of a high risk person who would die otherwise. \begin{equation*}B\end{equation*} can be thought of as  the “relative benefit” in preventing mortality of receiving a predispensed course of antivirals for a high risk person who takes it, compared to receiving a non-predispensed course. \begin{equation*}B\end{equation*} captures the benefit of early vs. delayed treatment; however, \begin{equation*}B\end{equation*} also may be reduced to the extent that a predispensed course is taken for non-influenza illness, in which case it cannot save a life.  Thus \begin{equation*}B\end{equation*} exceeds one to the extent that early treatment is better than delayed treatment, but it is decremented in proportion to the probability that the course is wasted before it is needed.

Let \begin{equation*}L\end{equation*} be the total number of people in the general population who would die during the epidemic and whose lives would be saved if they received antivirals upon demand during the course of the epidemic.  Since the total number of people in the total  population who would die during the epidemic and whose lives would be saved if they received antivirals during the course of the epidemic is \begin{equation*}p_dp_s\end{equation*} , clearly \begin{equation*}L\leq p_dp_s\end{equation*} .  We make additional assumptions about how the probability of receiving antivirals behaves if demand exceeds supply; these are made explicit in Appendix A.

TABLE 1: Parameters of the model, as a proportion of the total population (including high-risk and general populations)

**Table:** 

**Parameter **	**Definition**
\begin{equation*}T\end{equation*}	Supply of antivirals, before predispensing, as a fraction of total population size
\begin{equation*}D,D'\end{equation*}	Number of antiviral courses used by the general population ( \begin{equation*}D\end{equation*} ) or total population ( \begin{equation*}D'\end{equation*} ) if no supply constraint, as fraction of the total population size
\begin{equation*}L\end{equation*}	Total number of lives in the general population that would be saved under no predispensing, if no supply constraint
\begin{equation*}q\end{equation*}	Proportion of persons in the high risk group to whom predispensing of one course is considered, hence also the number of courses considered for predispensing, as a fraction of the total population
\begin{equation*}p_s\end{equation*}	Probability that a course of antivirals obtained during the epidemic would save a life of a person who would die otherwise, absent any predispensing
\begin{equation*}B\end{equation*}	Relative benefit (in terms of probability of saving life) for a high risk person to be treated early (with a predispensed course) compared to being treated with a non-predispensed course. Thus \begin{equation*}p_SB\end{equation*} is the probability that a predispensed course of antivirals would save a life of a high risk person who would die otherwise
\begin{equation*}p_d\end{equation*}	Death rate from the epidemic in the whole population, if no antivirals were used
\begin{equation*}R\end{equation*}	Ratio between the death rate in the high risk group and the general population.

With this notation in place, we can define the conditions under which it is advantageous to predispense 1 course each to a proportion \begin{equation*}q\end{equation*} of the population.  Our main result is the following:

MAIN RESULT: Predispensing saves more lives than not predispensing when any of the following conditions hold:

 (i)                   Supply of antivirals exceeds demand even after predispensing, and \begin{equation*}B>1\end{equation*} .

 (ii)                 Demand \begin{equation*}D\end{equation*} in the general population exceeds supply even without predispensing, and the following conditions are met:      

\begin{equation*}B> \frac{T}{D}\end{equation*} and \begin{equation*}R>\frac{1}{D(B-T/D)}\end{equation*}

 (iii)         Supply exceeds demand without predispensing, but after predispensing, demand exceeds supply, and the following conditions are met:           

\begin{equation*}B> \frac{T}{D}\end{equation*} and \begin{equation*}R>\frac{D+q-T}{Dq(B-1)}\end{equation*} .

(iv)    The above results show that even if demand cannot be predicted, predispensing is advantageous when

\begin{equation*}B>1\end{equation*} and \begin{equation*}R>\frac{1}{T(B-1)}\end{equation*}

The justification of this result and the assumptions underlying it are presented in Appendix B.  Condition (iv) tells us when a group is at high enough risk that it is worth predispensing to them even if we have no idea of the expected antiviral demand.

This result can be seen graphically in Figure 1, which considers a hypothetical case in which there is a supply adequate for \begin{equation*}T=0.2\end{equation*} of the population, and predispensing to \begin{equation*}q=3\%\end{equation*} of the total population is under consideration.  Also, we assume that treatment of a high-risk person with a predispensed course is twice as likely to be life-saving as treatment with a non-predispensed course, because predispensing allows earlier initiation of treatment ( \begin{equation*}B=2\end{equation*} ). The far left side shows low levels of demand, in which there is no harm to predispensing, so predispensing to any group may be beneficial.  At the far right, competition for antivirals is strong, so it is beneficial to predispense even to a group that gets modest benefit from antivirals.  In the middle, when demand is similar to supply, it is beneficial to predispense only to groups that benefit disproportionately from antivirals ( \begin{equation*}R>5\end{equation*} ).  

**Figure:**
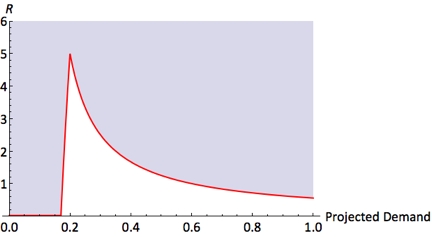
*FIGURE 1: Predispensing is beneficial (shaded area) when   exceeds a certain threshold that depends on the projected demand.  Here a supply adequate for   of the population is assumed, and predispensing to   of the total population is under consideration.  Also, we assume that treatment of a high-risk person with a predispensed course is twice as likely to be life-saving as if treatment is with a non-predispensed course, because predispensing allows earlier initiation of treatment (  ).  For low projected demand (below  ), there is no harm to predispensing, and predispensing even to groups that will gain below-average benefit from antivirals is better than no predispensing.  For very high projected demand, (   near 1), predispensing is beneficial even for very modest values of  , because competition for antivirals is strong, and predispensing has a relative benefit compared to acquisition by ill persons.  For projected demand comparable to the supply (  ), predispensing is valuable only for groups for whom antivirals are considerably more valuable than the general population, because each course predispensed comes at the expense of someone else who could use it, yet it does not strongly increase the chance that a high-risk individual gets treated (since that individual would likely receive a course anyway without predispensing). *

Figure 2 shows an equivalent plot for the case where there is no intrinsic benefit to predispensing, because a predispensed course is no more likely to save a life than a non-predispensed one.  Here, again there is an advantage to predispensing to even moderate risk groups if demand is high or low, but if demand is similar to supply, then predispensing is not advantageous.   For reasons elaborated below, we consider it likely that this is an extreme case and that in reality \begin{equation*}B>1\end{equation*} .  

**Figure:**
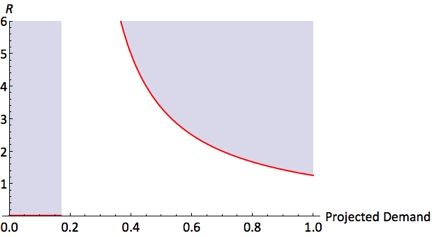
*FIGURE 2: If predispensed courses are no more likely to be lifesaving than non-predispensed courses taken by the same person, the conditions for benefits of predispensing are more restrictive.  Parameters are as in figure 1, except that treatment of a high-risk person with a predispensed course is equally likely to be life-saving as treatment is with a non-predispensed course (  ).   In particular, in this situation if demand=supply, it is never beneficial to predispense a course, since it may go to someone who does not need it, and it confers no benefit purely from being predispensed*

Finally, we note that while it is beneficial to predispense antivirals if either of the conditions in the Main Result are met, it is not true in general that the benefit increases with the quantity of antivirals predispensed. Under certain conditions it would be better to predispense to a subset of the high-risk population.  This is discussed further in Appendix C, where a simple criterion is given ensuring that each successive predispensed dose increases the net benefit, provided that the quantity of antivirals predispensed is not too large.

**Interpretation of these Conditions** 

Predispensing is most advantageous for groups that have the highest mortality rate (high \begin{equation*}R\end{equation*} ) and that benefit most from predispensed antivirals compared to non-predispensed ones (high \begin{equation*}B\end{equation*} ).  These factors capture the demand-independent aspect that it is useful to position antivirals with individuals who can be most helped by having them close at hand, rather than getting them after they become ill.  A second benefit of predispensing is to ensure access of antivirals to those who will benefit the most from them.  This benefit is greatest when demand is very high, because in such circumstances the high-risk individuals are very likely to need antivirals, but not to get them.  Thus the benefits of predispensing are greatest for larger values of \begin{equation*}R\end{equation*} , \begin{equation*}B\end{equation*} , and \begin{equation*}D/T\end{equation*} .  The costs of predispensing – lost opportunities for individuals who do not receive predispensed courses – are zero when demand is low enough (as all who need the antivirals can receive them) then increase, but not as fast as the benefits.  Qualitatively, then, predispensing is most advantageous when demand is very high or very low, and least likely to be advantageous when demand just exceeds supply.  Even there, however, if the benefit of a predispensed course is significantly larger than that of a non-predispensed course, predispensing is valuable for individuals who can benefit more from treatment than the general population.

**Risks of Predispensing**

The major risk of predispensing is that courses that would otherwise be life-saving (when used from the stockpile) will be predispensed, then not used by someone for whom they would be more likely to be life-saving.  We can consider what has been written so far from another perspective: this risk is most acute when demand is near supply ( \begin{equation*}T\approx D\end{equation*} ) , when individuals not in the group to be predispensed would benefit from treatment by predispensed antivirals which won’t be used, and when predispensing and consequent early use are not very beneficial (reducing \begin{equation*}B\end{equation*} ).

The idea of predispensing was previously considered in the context of pre-pandemic sale of antiviral “Medkits,” special packaging of oseltamivir or zanamivir for home storage to be saved until a pandemic occurred.  A number of concerns were raised concerning such Medkits, resulting eventually in a lack of approval by the Food and Drug Administration (www.fda.gov/ohrms/dockets/ac/08/minutes/2008-4385m1-final.pdf).  The risk of inappropriate use or wastage was prominent among these concerns.  One key difference between pre-pandemic distribution of antivirals and predispensing just prior to an anticipated wave of pandemic influenza is that the time frame for wastage is much shorter in the present case – only the time from when the antivirals are predispensed until the time when the individual becomes infected with influenza and needs them.  In the pre-pandemic setting, this might be years, while in the present case – considering only the upcoming epidemic wave – the time scale is only a matter of a few months.  This consideration makes wastage a less compelling argument.  Thus, we expect that \begin{equation*}B\end{equation*} will primarily reflect the advantage of early vs. delayed treatment (for which there is some evidence, although not for mortality) [6]. 

It is also worth mentioning that unlike the situation with antibacterial medications – which can promote drug resistance even when used to treat non-bacterial infections, because of their effect on bystander flora [7], anti-influenza drugs do not promote resistance when used to treat non-influenza infections.  Thus, the concern that predispensing could lead to inappropriate use and thereby to resistance may have some basis (if real influenza infections are treated suboptimally) but is not in the same category as the equivalent risk for antibacterial drugs.

**Implications for Decision Making**

On current evidence, we believe that there are likely to be groups in the population for whom the relative risk of death or of other severe outcomes, such as hospitalization, substantially exceeds 1.  These groups include pregnant women[5] and some of the other high-risk groups http://www.cdc.gov/h1n1flu/recommendations.htm.   We believe it is also plausible to expect that predispensed antivirals are more likely to be life-saving (or to prevent hospitalizations or other severe outcomes) than those that are not predispensed ( \begin{equation*}B>1\end{equation*} ), because wastage is relatively unlikely given the short time frame between when predispensing could occur and when the main wave of the epidemic is likely to come, and because early treatment is likely to have benefits in preventing progression to more severe disease.  Precise data will not be available to evaluate this assumption in time for decision making, so educated opinion will be the only basis for decision making.  We have found (E. Goldstein et al., Unpublished Data) from studies of a US city that approximately 20% of individuals with confirmed H1N1pdm infection took antivirals within 48 hours of infection.  Predispensing should be able to increase this proportion to well above 40%.  We proceed on the assumption that antivirals are twice as likely to save a life if predispensed than if received by normal channels.

One additional assumption that should be highlighted is that, as used in the absence of predispensing, antivirals are equally likely to save the life of a treated member of the general population who would die without treatment, and of a treated member of the high-risk group who would die without treatment.  If treatment is less effective among members of high risk groups who would die without treatment, then the benefits of predispensing are reduced; if it is more effective, they may be increased.  

Under these assumptions, then predispensing to members of groups at very high risk of fatal outcome (say at least 5 times the population average) will be beneficial, for almost any level of demand, if supply is adequate for 20% of the population (a reasonable estimate given current US stockpiles and the need to reserve some for health care worker prophylaxis).  Moreover, if demand is either less than or considerably more than supply of antivirals, predispensing to groups with relative risk of death larger than 1 but less than 5 will be beneficial. While we do not know how to predict demand, we believe that the first two conditions are likely to occur for pregnant women and other risk groups, especially for those with mobility or other impairments that make it difficult for them to obtain rapid medical attention.  One reason to expect that demand may be less than the antiviral supply is the experience in New Zealand, one country in the Southern Hemisphere for which population-based general practitioner consultation rates are available.  In that country, where the winter epidemic appears to be concluding, only approximately 1% of the population has consulted their GP for influenza-like illness (http://www.moh.govt.nz/moh.nsf/indexmh/influenza-a-h1n1-update-138-180809).  This may indicate that attack rates will be relatively low in the autumn wave in the Northern Hemisphere, though there are many differences between New Zealand and Northern Hemisphere countries, including over-the-counter availability of oseltamivir in New Zealand, which may reduce consultation rates in New Zealand. 

At present there are no published statistics on which groups are at highest risk of severe outcome (hence most likely to have high \begin{equation*}R\end{equation*} ), except pregnant women (4) .  Such data are urgently needed to prioritize many types of interventions, including vaccination and antiviral predispensing.  Once such data (or estimates) are available, we would recommend predispensing to all members of groups at very high risk of death (say more than 10), on the grounds that this will be beneficial except for the rather unlikely case of no relative benefit ( \begin{equation*}B=1\end{equation*} ) and supply closely matching demand ( \begin{equation*}T\approx D\end{equation*} ).  We would recommend strong consideration of predispensing for groups with relative risk of death greater than 3-4, on the grounds that this will surely be beneficial, as long as \begin{equation*}B\geq 2\end{equation*} , for almost any level of demand.  We would, however, recommend that the total proportion of the stockpile predispensed be limited (perhaps to 20% of the stockpile or less), first because there is the possibility that risk groups will change if the virus changes, and second for the reason described in the next paragraph.

In the setting of a rapidly emerging autumn wave of pandemic influenza in developed countries of the Northern Hemisphere that already possess significant antiviral stockpiles, predispensing of a portion of these stockpiles to individuals at high risk of severe outcome of infection may be a means to prevent death and other severe outcomes by improving the efficiency of use of a limited stockpile.  

The conditions defined in this paper guarantee that predispensing is beneficial in terms of decreasing the mortality provided certain assumptions about temporal patterns of antiviral distribution hold. The most flexible assumption we require is assumption b) in Appendix A. This assumption may be violated under the following scenario: antiviral supply nears depletion and only the most severe cases get antivirals. To deal with such a scenario, we recommend to set aside a certain quantity of antivirals for safekeeping. Our conditions would then need to be evaluated against the rest of the stockpile.

In summary, we urge rapid identification of the groups at highest risk of death, long-term intensive care stays, or hospitalization, using the best available clinical data, and we urge public health bodies, professional organizations and providers to encourage members of the highest risk groups to obtain antivirals as soon as possible for use during an autumn pandemic wave.

**Acknowledgments**

We thank Richard Hatchett for helpful discussions.  This work was funded by the US National Institutes of Health Models of Infectious Disease Agent Study Cooperative Agreements 5U01GM076497 and 1U54GM088588 to ML for the Harvard Center for Communicable Disease Dynamics, and by a RAPIDD postdoctoral fellowship (JCM).

**Competing Interests**

ML discloses consulting fees from the Avian/Pandemic Flu Registry (Outcome Sciences), funded in part by Roche.** ** All other authors declare no competing interests.

**APPENDIX:**

**A.  Justification of the Main Result**

In this Appendix we derive the main result by calculating or finding a bound on the number of lives saved by antiviral treatment in the high risk group (a proportion \begin{equation*}q\end{equation*} ) and in what we term the “general population,” which is the total population without the high risk individuals, a proportion \begin{equation*}1-q\end{equation*} .  

Let us first list the assumptions we are making about the temporal patterns of antiviral demand used in out Main Result:

 a)    For condition (ii) to imply the benefit of predispensing, we need that among the people who receive antivirals, the proportion of them who would die otherwise doesn’t change in time.

 b)    For conditions (iii) and (iv) to imply the benefit of predispensing, we need that among the people who receive antivirals, the proportion of them who would die otherwise doesn’t increase in time. 

 c)    For condition (i), no additional assumptions are needed.

Condition b) is more flexible than a) and can be interpreted as follows: consider persons infected during the epidemic. As time progresses, the proportion of them who will require antivirals may increase due to panic, etc. - suppose the increase is by a factor of \begin{equation*}X\end{equation*} . The people who would die without antivirals are the severe cases and the proportion of them who receives antivirals (given availability) is probably large - thus even if it increases it time, we assume that the increase is by a factor of at most \begin{equation*}X\end{equation*} .

There are four conditions to consider: 

**Condition (i): Supply exceeds demand even after predispensing  and **  

In this case all persons in the general population who demand antivirals would get them under the no predispensing scenario – thus predispensing would result in no loss of lives in that category compared to no predispensing. Since  , persons to whom antivirals were predispensed are more likely to survive under predispensing than through acquiring antivirals during the epidemic. Moreover no all among them who need antivirals would necessarily acquire them during the epidemic – thus predispensing is clearly beneficial.

**Condition (ii): In the absence of predispensing, demand exceeds supply,**

To fix notation, let \begin{equation*}D'\geq D\end{equation*} be the demand for antivirals in the total population in the absence of predispensing.

Among the \begin{equation*}q\end{equation*} individuals in the high risk group, \begin{equation*}qRp_d\end{equation*} of them would die without antivirals. Out of those, \begin{equation*}qRBp_dp_s\end{equation*} will be saved by predispensed antivirals. Under no predispensing, not all of those \begin{equation*}qRp_d\end{equation*} people may demand antivirals, and among those who do, a fraction \begin{equation*}\frac{T}{D'}\leq \frac{T}{D}\end{equation*} of them would get antivirals, so \begin{equation*}qRp_dp_s\frac{T}{D'}\end{equation*} lives would be saved (Table 2). 

TABLE 2: Numbers of lives saved in the general population and high risk group under no predispensing vs. predispensing, Case 1 (demand exceeds supply)

**Table:** 

** **	**No predispensing**	**Predispensing**
General population	at most \begin{equation*}\frac{T}{D'}L\end{equation*}	at least \begin{equation*}\frac{T-q}{D}L\end{equation*}
High-risk group	at most \begin{equation*}\frac{T}{D'}qRp_dp_s\end{equation*}	\begin{equation*}qRBp_dp_s\end{equation*}

Turning now to the general population, if all the demand was met, the total number of lives saved without predispensing would be \begin{equation*}L\end{equation*} .  However, given scarcity, only \begin{equation*}\frac{T}{D'}L\end{equation*} lives are saved (Table 2), because the remaining individuals do not receive antivirals.  If antivirals are predispensed, the remaining demand is \begin{equation*}D\end{equation*} and the supply is \begin{equation*}T-q\end{equation*} . Thus a fraction \begin{equation*}\frac{T-q}{D}\end{equation*} of  the demand in the general population is met, and the number of lives saved is thus \begin{equation*}\frac{T-q}{D}L\end{equation*} (Table 2).   

Using these figures (summarized in Table 2), there is a net gain in lives saved if the total number saved under predispensing exceeds that saved without predispensing, that is, if 

                                     (A1) $L\frac{T - q}{D} + qRBp_d p_s \geq L\frac{T}{D'} + qRp_d p_s \frac{T}{D'}$ .

Rearranging and dividing by \begin{equation*}q>0\end{equation*} , we obtain the condition $L\left(\frac{1}{D} + \frac{T}{q}\left[ \frac{1}{D'} - \frac{1}{D}\right]\right) \leq Rp_d p_s B - Rp_d p_s \frac{T}{{D'}}$ .  Using the facts that \begin{equation*}D'\geq D\end{equation*} and \begin{equation*}L \le p_s p_d\end{equation*} , a sufficient condition is then 

                                    (A2) \begin{equation*}\frac{1}{D} \le R(B - T/D)\end{equation*}

which is condition (ii) so long as \begin{equation*}B>T/D\end{equation*} .

**Condition (iii)**

The argument is similar to the previous case. 

Among the high-risk group, without predispensing nearly all or all demand will be satisfied (there may be some competition if $D \le T \le D'$ ), so at most \begin{equation*}qRp_dp_s\end{equation*} lives will be saved.  With predispensing, the situation will be as above.

Among the general population, without predispensing, at most \begin{equation*}L\end{equation*} lives will be saved.   With predispensing, however, the situation will be as above in Condition (ii).  This condition is summarized in Table 3.  Repeating the comparison of equation (A1) for the entries in Table 3, we obtain Condition (iii).

TABLE 3: Numbers of lives saved in the general population and high risk group under no predispensing vs. predispensing, Case 3 (supply exceeds demand before predispensing, but not after)

**Table:** 

** **	**No predispensing**	**Predispensing**
General population	at most \begin{equation*}L\end{equation*}	at least \begin{equation*}\frac{T-q}{D}L\end{equation*}
High-risk group	at most \begin{equation*}qRp_dp_s\end{equation*}	\begin{equation*}qRBp_dp_s\end{equation*}

**Condition (iv) no assumptions on demand.**

If \begin{equation*}B>1\end{equation*} and \begin{equation*}R>\frac{1}{T(B-1)}\end{equation*} , then depending on whether \begin{equation*}D \leq T-q\end{equation*} , \begin{equation*}T-q \leq D \leq T\end{equation*} , or \begin{equation*}T<D\end{equation*} , one of the above conditions applies.     

**B.  Formal description of the parameters ,   and  .**  

Suppose that for every individual who is either a member of the high risk group \begin{equation*}G=h\end{equation*} or the total population \begin{equation*}G=w\end{equation*} , we can define an outcome \begin{equation*}O(T)\end{equation*} that depends on the (possibly counterfactual) treatment \begin{equation*}T\end{equation*} received, where either \begin{equation*}O=d\end{equation*} (died) or \begin{equation*}O=s\end{equation*} (survived) and either \begin{equation*}T=U\end{equation*} (untreated), or \begin{equation*}T=S\end{equation*} (treated from the stockpile), or \begin{equation*}T=P\end{equation*} (treated from a predispensed course, and that course has not been previously wasted).  Then 

\begin{equation*} \begin{array}{l} p_D = \Pr [O(U) = d|G = w] \\ p_S = \Pr [O(S) = s|O(U) = d] = \Pr [O(S) = s|O(U) = d,G = w] = \Pr [O(S) = s|O(U) = d,G = h] \end{array} \end{equation*}

\begin{equation*} \begin{array}{l} R = \frac{{\Pr [O(U) = d|G = h]}}{{\Pr [O(U) = d|G = w]}} \\ B = \frac{{\Pr [O(P) = s|O(U) = d,G = h]}}{{\Pr [O(S) = s|O(U) = d,G = h]}} \\ \end{array} \end{equation*}

We are interested in the probability that treatment (nonpredispensed) will save a life in the total population, which is 

\begin{equation*} p_d p_s = \Pr [O(U) = d,O(S) = s|G = w] \end{equation*} ,

in the probability that treatment without predispensing will save a life in the high-risk group, which is 

\begin{equation*} \begin{array}{l} p_D p_S R = \Pr [O(U) = d|G = h]\frac{{\Pr [O(U) = d|G = h]}}{{\Pr [O(U) = d|G = w]}}\Pr [O(S) = s|O(U) = d,G = h] \\ = \Pr [O(S) = s,O(U) = d|G = h] \\ \end{array} \end{equation*}

and in the probability that a high risk person who received a predispensed course would thereby have his life saved, which is 

\begin{equation*} p_D p_S RB = \Pr [O(T) = s,O(U) = d|G = h] \end{equation*}

**C. When does the benefit increase with the number of courses predispensed?**

While predispensing to high risk groups is beneficial compared to no predispensing, if condition (iv) holds, it is not true in general that the benefit of predispensing increases with the quantity of antivirals predispensed. Consider for example one high risk group whose size equals (or surpasses) the antiviral supply.  As we keep on predispensing, coverage level for the rest of the population goes to 0  while the relative risk of dying in the high risk group stays uniformly bounded. Thus at some point equation (iv) will be violated (with the new coverage level and the relative risk), and in fact it is not advisable to predispense the whole supply to the group under no a-priori assumptions on the demand \begin{equation*}D\end{equation*} . However the have the following simple criterion:   

BENEFIT INCREASES WITH THE AMOUNT PREDISPENSED: 

Suppose the relative risk of dying $R \ge 1$ , and assumption b) in Appendix A holds. The benefit of predispensing is guaranteed to increase with quantity of antivirals predispensed as long as the total quantity \begin{equation*}q\end{equation*} of antivirals predispensed obeys

                           (C1) $\frac{{T - q}}{{1 - q}} \ge \frac{1}{{R(B - 1)}}$

To show this, let the quantity \begin{equation*}q\end{equation*} of antivirals predispensed obey the inequality (C1). To show that the benefit of predispensing was increasing, take any intermediate quantity \begin{equation*}q_I\leq q\end{equation*} . We need the show that equation (C1) was still true after predispensing \begin{equation*}q_I\end{equation*} courses of antivirals. Note that after this predispensing, the coverage level and the relative risks have changed. The coverage level became

                           (C2) \begin{equation*} T_I = \frac{{T - q_I }}{{1 - q_I }} \ge \frac{{T - q}}{{1 - q}} \ge \frac{1}{{R(B - 1)}} \end{equation*}

Also, as a result of predispensing, we've removed some individuals with the risk of dying higher than the one for the general population. Thus the probability of dying for an average individual left after predispensing is lower than the one before predispensing; hence the relative risks of the high risk groups have only increased. Since equation (C2) for the new coverage level holds with original relative risks, condition (iv) in our Main Result holds with the new coverage levels and relative risks (after predispensing \begin{equation*}q_I\end{equation*} courses of antivirals). This implies that predispensing \begin{equation*}q-q_I\end{equation*} courses after predispensing the initial \begin{equation*}q_I\end{equation*} courses would be beneficial.

## References

[ref-3615491467] Miller MA, Viboud C, Balinska M, Simonsen L. The signature features of influenza pandemics--implications for policy. N Engl J Med. 2009 Jun 18;360(25):2595-8. Epub 2009 May 7. 19423872 10.1056/NEJMp0903906

[ref-3413670683] Centers for Disease Control and Prevention, H1N1 Flu (Swine Flu): Resources for Child Care Programs, Schools, Colleges, and Universitieshttp://www.cdc.gov/h1n1flu/schools/

[ref-1842047783] Miller MA, Viboud C, Olson DR, Grais RF, Rabaa MA, Simonsen L. Prioritization of influenza pandemic vaccination to minimize years of life lost. J Infect Dis. 2008 Aug 1;198(3):305-11. 18558871 10.1086/589716 PMC3206321

[ref-2319176823] Dushoff J, Plotkin JB, Viboud C, Simonsen L, Miller M, Loeb M, Earn DJ. Vaccinating to protect a vulnerable subpopulation. PLoS Med. 2007 May;4(5):e174. 17518515 10.1371/journal.pmed.0040174 PMC1872043

[ref-1860550007] Jamieson DJ, Honein MA, Rasmussen SA, Williams JL, Swerdlow DL, Biggerstaff MS, Lindstrom S, Louie JK, Christ CM, Bohm SR, Fonseca VP, Ritger KA, Kuhles DJ, Eggers P, Bruce H, Davidson HA, Lutterloh E, Harris ML, Burke C, Cocoros N, Finelli L, MacFarlane KF, Shu B, Olsen SJ; Novel Influenza A (H1N1) Pregnancy Working Group. H1N1 2009 influenza virus infection during pregnancy in the USA. Lancet. 2009 Aug 8;374(9688):451-8. Epub 2009 Jul 28. 19643469 10.1016/S0140-6736(09)61304-0

[ref-709764055] Burch J, Corbett M, Stock C, Nicholson K, Elliot AJ, Duffy S, Westwood M, Palmer S, Stewart L. Prescription of anti-influenza drugs for healthy adults: a systematic review and meta-analysis. Lancet Infect Dis. 2009 Aug 7. [Epub ahead of print] 19665930 10.1016/S1473-3099(09)70199-9

[ref-2022504715] Lipsitch M, Samore MH. Antimicrobial use and antimicrobial resistance: a population perspective. Emerg Infect Dis. 2002 Apr;8(4):347-54. Erratum in: Emerg Infect Dis 2002 May;8(5):540. 11971765 10.3201/eid0804.010312 PMC2730242

